# The Influence of Living Conditions on Self-Rated Health: Evidence from China

**DOI:** 10.3390/ijerph18179200

**Published:** 2021-08-31

**Authors:** Jiafeng Gu, Xing Ming

**Affiliations:** 1 Institute of Social Science Survey, Peking University, Beijing 100871, China; 2 School of Sociology and Political Science, Shanghai University, Shanghai 200444, China; coclover@shu.edu.cn

**Keywords:** living conditions, self-rated health, housing, logistic regression, China

## Abstract

Despite growing attention to living conditions as a social determinant of health, few studies have focused on its diverse impacts on self-rated health. Using data from the China Family Panel Study in 2018, this study used logistic regression analysis to examine how living conditions affect self-rated health in China, finding that people cooking with sanitary water and clean fuel were more likely to report good health, and that homeownership was associated with higher self-rated health. The self-rated health of people living in high-quality housing was lower than that of people living in ordinary housing, and people living in tidy homes were more likely to report good health. The findings suggest that the link between multiple living conditions and self-rated health is dynamic. Public health policies and housing subsidy programs should therefore be designed based on a comprehensive account of not only housing grade or income status, but also whole dwelling conditions.

## 1. Introduction

Living conditions and health are of broad interest to health researchers, policymakers, and the general public. An increasing number of studies have shown that living conditions are fundamental social determinants of health [1]. Living conditions have direct or indirect effects on physical and mental health [2]. Improvements in housing quality are conducive to improving the mental health of residents [3]. Poor living conditions can lead to the spread of infectious diseases, chronic diseases, nutritional deficiencies, and mental health problems [4,5]. In addition, living conditions at the community level also have an impact on health [6,7]. Communities close to major roads, bus stops, and airports adversely affect the health of residents in the community [8,9,10]. However, due to the complexity of living conditions and the diversity of living spaces, the impact mechanism of living conditions on health has not been clarified, and a general consensus has yet to be reached [11]. The relationship between living conditions and health is by no means clear at a glance. The World Health Organization recommends that information on how individuals perceive their own health should be collected in population-based studies [12]. Self-rated health status, reflecting both physical and mental health as well as well-being, is considered a sensitive and holistic measure of overall health [13,14]. At present, the proportion of people’s self-rated health depends on the influence of living conditions and is not completely scientifically explained.

The general aim of this study was to determine the connections between people’s self-rated health and the environment in which they live. Cognition about the connections between certain living conditions and self-rated health can be crucial for the possibility of environment and housing programming for healthier living. As the largest developing country, China has the largest population in the world and is actively implementing measures for the renovation and governance of residential space to improve living conditions as part of the implementation of a healthy China strategy. Thus, China is a very important area for research on housing and health. This study used data from the China Family Panel Study in 2018 to examine the impact of living conditions on residents’ self-rated health and to determine the impact mechanism. This paper is arranged as follows: Section 2 presents the literature review and hypothesis development; Section 3 presents the data, samples, and methods; Section 4 presents the results; Section 5 is the discussion; and the last section is the conclusion.

## 2. Literature Review and Hypothesis Development

Poor living conditions are often associated with poor health [15,16]. Substandard housing including lack of safe drinking water, sanitary water for cooking, effective garbage disposal devices, overcrowding, and poor ventilation has been confirmed to be closely related to the spread of respiratory infectious diseases [17,18]. Therefore, international organizations, including the World Health Organization and the World Bank, have called for improved health by improving living conditions [19,20]. Governments of various countries are also actively improving living conditions to protect residents’ health [7]. However, a large gap still exists in our knowledge of the links and pathways between living conditions and health status [21]. Research on living conditions and health in Western countries started early and has been relatively comprehensive so far [22,23], having mainly studied the impact on health from housing acquisition methods, housing internal and external environments, and homelessness [24,25,26,27]. However, there is a lack of such research in China, the world’s second largest economy, which is not commensurate with the current world pattern. As the largest developing country in the world, China’s experience in housing reform, living condition improvement, and health improvement hold important implications and value for the vast number of developing countries [28,29]. Therefore, empirical testing of relevant hypotheses about the relationship between living conditions and health in China has important theoretical and practical value.

Water quality has an important impact on human health [30]. Water participates in the transportation and metabolism of various substances in the human body and maintains blood circulation, breathing, digestion, absorption, secretion, excretion, and other physiological activities [31]. Access to safe drinking water is important for health. There is currently no uniform definition of what is meant by “safe” water, and the standards for drinking water safety in various countries are not uniform. However, all countries in the world have a general consensus on the health of drinking water; that is, drinking raw water directly is not conducive to human health [32]. River water, stream water, well water, reservoir water, and other water bodies contain various harmful bacteria, microorganisms, and zoonotic parasites to varying degrees. In particular, the development of modern industry and agriculture has caused existing surface water to be polluted by factory wastewater [33]. In some remote areas of China, some people still drink raw water or cook with raw water today. After drinking raw water or cooking with raw water, it is easy to contract acute gastroenteritis, typhoid fever, dysentery, and parasitic infections [34]. Cooking of rice with raw water has also been identified as an important or potentially important exposure route to groundwater arsenic [35]. In general, drinking tap water is safe [36,37]. Tap water is taken directly from natural water sources (surface water and groundwater), and passes through a series of treatment processes, such as sedimentation, gelation, sand filtration, and disinfection, before being provided to users. People are also willing to pay for high-quality tap water [38]. Therefore, the expected self-rated health of people who cook with tap water and purified water is higher than that of people who cook with unfiltered water. Therefore, the following hypothesis is proposed:

**Hypothesis** **1.** *People cooking with sanitary water will have a higher self-rated health than those who cook with raw water.*

Cooking fuel is a component of indoor pollution [39]. Clean cooking has now emerged as a global concern because of the established negative health impacts associated with solid fuel cooking [21]. In Indonesia, household air pollution from solid fuel combustion is linked to 165,000 premature deaths each year [40]. Studies have confirmed that using solid fuels such as coal, straw, and firewood as the main life fuel significantly increases the risk of a variety of diseases such as acute respiratory infections, heart disease, stroke, and lung cancer [41,42,43]. The burning of solid fuels for indoor heating and cooking is the primary risk factor for chronic obstructive pulmonary disease (COPD) [44]. In contrast, using cleaner cooking fuels can significantly improve physical and mental health [45]. Compared to women whose households cook with traditional fuels such as wood/straw, women whose households cook with clean fuels such as liquefied petroleum gas have a significantly lower probability of chronic or acute diseases and are more likely to report better health [46]. Therefore, the following hypothesis is proposed:

**Hypothesis** **2.** *People cooking with clean fuels have higher self-rated health than those who cook with traditional fuels.*

As a fixed asset, a house is a symbol of social status, and homeownership gives homeowners a greater sense of comfort derived from favorable economic status, which is good for their health [47,48,49]. Partial ownership or even no housing ownership will induce inferiority and anxiety, which will negatively affect health [50]. Relevant survey data in South Korea show that compared with those who own houses, renters are more likely to have depressive symptoms and have poorer self-rated health, especially when renters face high rental costs, which negatively affect their health [51]. According to the Longitudinal Survey of the Office of Census and Surveys (OPCS) in the United Kingdom between 1981 and 1989, the mortality rate of men in rented housing was 22% higher than that of men in self-owned housing, and 32% higher in women [52]. Therefore, the following hypothesis is proposed:

**Hypothesis** **3.** *The self-rated health of people who own houses with full ownership rights will be higher than that of people who do not own houses with full ownership rights.*

With the improvement of the people’s living standards, the housing conditions of the Chinese are constantly improving. According to data from the National Bureau of Statistics of China, from 1978 to 2019, the per capita housing area of urban residents increased from 6.7 square meters to 39.8 square meters, and the per capita housing area of rural residents increased from 8.1 square meters to 48. 9 square meters. However, since the 21st century, especially in the past 10 years, with the continuous improvement of China’s housing marketization, the degree of housing stratification has intensified in China [53]. Housing stratification refers to the emergence of a three-part pattern of housing proletarians, property owners, and wealthy people. It is not only the result of the polarization between the rich and the poor, but also the starting point of the polarization; it not only reflects the polarization between the rich and the poor, but can also exacerbate the polarization between the rich and the poor [54,55]. Housing stratification has produced a series of consequences, among which the impact on health has gradually attracted the attention of scholars [56].

Housing is a manifestation of economic strength [57]. Due to different economic statuses, different social groups have respective expectation for health. Richer people have higher health expectations, so they are always unsatisfied with their own health evaluations and will report low self-rated health [58]. People living in good housing conditions generally have to bear heavier mortgages and face greater pressure of mortgage foreclosure, which will have a negative impact on their health [59]. Moreover, increasing income does not always improve happiness or self-rated health [60]. People with a high income tend to report slightly worse health, which may be explained by higher education and higher level of concern for health [61]. Higher income classes living in high-quality housing are more strongly associated with obesity and are more likely to face various pressures and challenges, which will have a negative impact on their health [62]. A house that is too large will separate people from each other, resulting in fewer opportunities for family members to communicate and enhance family relationships, which is not good for health [63,64]. Accordingly, people living in ordinary houses such as bungalows, courtyards, and unit houses should have a higher evaluation of their own health, while people living in high-quality houses such as villas, townhouses, and small buildings should have lower evaluations of their own health. Therefore, the following hypothesis is proposed:

**Hypothesis** **4.** *The self-rated health of people living in high-quality housing will be lower than that of people living in ordinary housing.*

The neater the home, the cleaner and more comfortable an environment that can be created, which helps relieve depression. Living in an overcrowded and messy space can have a negative impact on health [65]. Studies show that people with clean houses are healthier than people with messy homes because household dust is an important reservoir of indoor allergens [66,67]. House cleanliness has been found to be even more a predictor of physical health than neighborhood walkability [68]. It is obvious that a messy environment hugely impacts a person’s life, both mentally and physically. A clean family environment will produce less family pressure, which could impact positively eating behaviors and metabolism [69]. Therefore, the following hypothesis is proposed:

**Hypothesis** **5.** *The self-rated health of people with tidy homes is higher than that of people with untidy homes.*

## 3. Data, Samples, and Methods

### 3.1. Data and Samples

The data used in this study were obtained from the China Family Panel Studies (CFPS), which is a nationwide, comprehensive longitudinal tracking survey conducted by the Institute of Social Science Survey of Peking University. By tracking and collecting data at three levels of individuals, families, and communities, it reflects the changes in China’s society, economy, population, education, and health. The CFPS sample covers a population of 25 provinces/cities/autonomous regions in China, except Hong Kong, Macau, Taiwan, Xinjiang, Tibet, Qinghai, Inner Mongolia, Ningxia, and Hainan. The population of these 25 provinces/cities/autonomous regions accounts for approximately 95% of the total population of the country (excluding Hong Kong, Macau, and Taiwan). The CFPS employs a multistage probability sample drawn using implicit stratification, and the CFPS sample can be regarded as a nationally representative sample. The survey used computer-assisted personal interviews to collect the data. The CFPS survey questionnaire has four main questionnaire types: community, family, adult, and child. To date, five follow-up surveys (CFPS2012, CFPS2014, CFPS2016, CFPS2018, and CFPS2020) have been conducted in addition to the 2010 baseline survey.

This study uses the family economic questionnaire data and adult questionnaire data from the 2018 follow-up survey because the data of CFPS2020 have not yet been released. Among them, there were a total of 14,241 observations in the family economic questionnaire and 32,669 observations in the adult questionnaire. The above two types of data were matched by personal family codes, and 32,669 observations were obtained. After deleting missing values, such as missing answers, inapplicable answers, refusal to answer, and inability to judge, there were 22,710 observations left, which is the total number of samples for this study.

### 3.2. Variables

In this study, the dependent variable “self-rated health” was used as the evaluation index of personal health status. The core explanatory variables are the living conditions as measured by five variables: whether the cooking water is sanitary, whether the cooking fuel is energy-efficient, who owns the house, the type of residential house, and the degree of cleanliness of the house. The type of residential house is divided into ordinary residences and high-quality residences. Ordinary residences include bungalows, Siheyuans, units, and others; high-quality residences include villas, townhouses, and small buildings. A bungalow refers to a one-story house with a reinforced concrete structure and a flat roof. Siheyuan, also known as Sihefang, is a traditional courtyard-style building in China. Its layout is a courtyard with houses on all sides, enclosing the courtyard from all sides in the middle. Units in China specifically refer to the type of residential buildings where each household has a private kitchen and toilet, which is equivalent to a western apartment. A villa refers to a garden residence built in the suburbs or scenic areas for recuperation, and is a residence for enjoying life. Townhouses are often located in the suburbs with convenient transportation with shared walls between neighbors, but single-family. A small building refers to a house with two or more floors, which is different from a bungalow. The control variables included community type, age, gender, marital status, education level, and income level. Community type includes two types: village committee and neighborhood committee. The villager committee is the grassroots mass autonomous organizations in rural areas and are the management institutions of rural communities in China. The neighborhood committee is a grassroots mass autonomous organization for self-management, self-education, and self-service of urban residents. The definitions of the variables are listed in Table 1. The correlation matrix is in Appendix A.

Multivariate logistic regression analysis was conducted to test each relationship using R3.5.2. Statistical significance was set at *p* < 0.05, and all tests were 2-tailed. The likelihood ratio test of the final multivariate logistic regression model against the null model and the χ^2^-statistic, as the difference between the −2log likelihoods of the null and final models, was conducted. Once the *p-*value was 0.001, it could be concluded that the final model outperformed the null model.

## 4. Results

The self-rated health of the respondents is presented in Table 2. In 2018, 67.3% of respondents reported good health (*n* = 15,294), while 32.7% of respondents reported poor health (*n* = 7416).

The results of the cross-contingency table show that people with a tendency toward good self-rated health were more likely to be people who cook with sanitary water and clean fuel than those who cook with unsanitary raw water and traditional fuel. Those who thought they were in good health were more likely to live in a fully owned house than those who lived in an incompletely owned house. However, there was no significant difference in the self-rated health of the groups living in high-quality and ordinary housing. For groups with tidy homes, the proportion of healthy people was significantly higher than the proportion of unhealthy people; for groups with untidy homes, the proportion of healthy people was significantly lower than that of unhealthy people. 

For the group whose community is the neighborhood committee, the proportion of people who self-rated as healthy was significantly higher than the proportion who self-rated as unhealthy; for the group whose community is the village committee, the proportion of people self-rated as healthy was significantly lower than those who self-rated as unhealthy. The proportion of men who self-rated as healthy was significantly higher than those who self-rated as unhealthy; the proportion of women who self-rated as healthy was significantly lower than those who self-rated as unhealthy. For groups with spouses, the proportion who self-rated as healthy was significantly lower than those who self-rated as unhealthy; for those without a spouse, the percentage who self-rated as healthy was significantly higher than those who self-rated as unhealthy. For the educated group, the proportion who self-rated as healthy was significantly higher than those who self-rated as unhealthy; for the uneducated group, the proportion who self-rated as healthy was significantly lower than those who self-rated as unhealthy. For groups with higher wages, the proportion who self-rated as healthy was significantly higher than those who self-rated as unhealthy; for groups with lower wages, the percentage who self-rated as healthy was significantly lower than those who self-rated as unhealthy.

Table 3 presents the regression results of multivariate logistic regression analysis. The Cox and Snell *R*^2^ value = 0.194 and ρ^2^ (Nagelkerke) = 0.191.

As shown in Table 3, for the explanatory variables, the average likelihood of self-rating as healthy among those using sanitary water for cooking was 1.16 times that of those using unsanitary water for cooking (OR = 1.159; 95% CI: 1.082–1.241). Thus, Hypothesis 1 was confirmed. The average likelihood of self-rating as healthy among people who used clean fuel for cooking was 1.14 higher than that of those who did not use clean fuel for cooking (OR = 1.141; 95% CI: 1.066–1.231). Thus, Hypothesis 2 is confirmed. The average probability of self-rating as healthy for those with complete housing ownership was 1.12 times that of those with incomplete housing ownership (OR = 1.115; 95% CI: 1.018–1.221). Thus, Hypothesis 3 is confirmed. The likelihood of those living in high-quality housing self-rating as healthy was only 90.8% that of those living in ordinary housing (OR = 0.908; 95% CI: 0.849–0.971). Thus, Hypothesis 4 is confirmed. The average likelihood of self-rating as healthy for a group with a tidy house is 1.26 times that of a group with a messy house (OR = 1.261; 95% CI: 1.186–1.342). Thus, Hypothesis 5 is confirmed.

For the control variables, there was no significant difference in self-rated health between those whose community was the neighborhood committee versus the village committee. Men were 1.45 times more likely to self-rate as healthy than women (OR = 1.452; 95% CI: 1.368–1.542). The educated group was 1.44 times more likely to self-rate as healthy than the uneducated group (OR = 1.438; 95% CI: 1.339–1.544). Those with higher wages are 1.56 times more likely to self-rate as healthy than those with lower wages (OR = 1.558; 95% CI: 1.451–1.672). Every increase in age by one year reduced the average likelihood of reporting good health by 3.6% (OR = 0.964; 95% CI: 0.962–0.966). The average probability of self-rating as healthy with a spouse was only 86.8% that of the group without a spouse (OR = 0.868; 95% CI: 0.797–0.945).

## 5. Discussion

The quality of drinking water is closely related to human health [31]. Ensuring the safety and salubriousness of drinking water is a major challenge in the sustainable development of human society. In modern industrial society, drinking water is threatened by various pollutants from production and life activities, and its impact on health is receiving increasing attention [30,33]. The results of this study show that people who cook with purified sanitary water have higher self-rated health than those who cook with unfiltered raw water. Water that has not undergone some sort of treatment process may contain animal feces and giardia, a parasite that can potentially cause diarrhea or vomiting [33,34]. In general, drinking tap water is safer and healthier. Tap water provides water that meets the standards after purification and disinfection, which is important for protecting people’s health [38]. Of course, drinking tap water poses health risks [70]. For example, a variety of toxic substances can still occur in tap water, even some carcinogens [71]. However, for most people, being able to drink tap water not only represents an improvement in the quality of drinking water, but also means that the modernization of daily life has been significantly improved. Therefore, it will produce a positive attitude toward life, and self-rated health will also be higher. 

Different cooking fuels have different effects on indoor pollution; therefore, their health effects are also different. The results of this study show that people who cook with clean fuels have a higher self-rated health than those who cook with traditional fuels. When solid fuels such as coal, straw, and firewood are used as the main living fuels, negative impacts on personal self-assessed health are likely. This is consistent with the findings of previous studies [44,45,47]. Cooking with clean fuel is very helpful to health, but the current challenge is that the proportion of cooking with clean fuel is not high enough. According to the WHO, in 2013 the percentage of people still relying on solid fuels for their daily cooking tasks was 79% in Sub-Saharan Africa, 63% in South-East Asia, and 40% in the Western Pacific region (rural China) [19]. Four billion people worldwide still lack access to clean, efficient, convenient, safe, reliable, and affordable cooking energy [20]. Our research further proves the necessity of accelerating the transition from traditional fuel cooking practices to clean fuel cooking practices.

There have been many studies of the relationship between housing ownership and residents’ health in Western countries [47,48,49,72]. However, there is a lack of relevant research in China. The results of this study show that the self-rated health of people who own their houses is better than that of renters or shared houses. This conclusion is consistent with the conclusions of related studies conducted in Western countries [47,48,49]. A similar situation exists in South Korea and Japan [53,54]. The positive impact of housing ownership on residents’ health has a certain universality. Of course, China has a certain degree of uniqueness in this issue. In China, people like to buy a house and enjoy the happiness of owning a house. In 2012, Southwestern University of Finance and Economics and the People’s Bank of China jointly issued a “Chinese Household Finance Survey Report”, which stated that China’s own home ownership rate was as high as 89.68%, far exceeding the world level of about 60% (65% in the United States, 70% in the United Kingdom, and 60% in Japan). However, as housing prices continue to rise, buying a house incurs high loans, making the homeowner a so-called “house slave” (*fangnu*) carrying a heavy mortgage loan, which is not conducive to their physical and mental health. “House slave” is a new term emerging along with the rapid rise of real estate prices in large cities in China, which is used to describe the living conditions of one social group buying houses with the help of large commercial loans that create great economic and psychological pressure. 

Defined from academic research, “house slave” refers to a family whose monthly debt repayment exceeds 50% of the family’s monthly income. Such conditions have affected the normal quality of family life due to their high debt ratio, which has a negative effect on the health of family members. Similar situations exist in the United States [73] and Europe [74]. Severe housing-cost burden was associated with an increase in the odds of childhood obesity in the United States [75]. In China, “house slaves” face a greater risk of repaying their loans, which will have a negative impact on their education expenditures and medical expenses and will significantly reduce the quality of family life even making people feel enslaved. These will have a negative impact on personal health [76]. Due to the different national conditions of each country, the impact of the housing cost burden may also be different. For such homeowners, the impact of house ownership and housing-cost burden on their health is worthy of further study.

Improved housing enhances human welfare in general, and has particular health benefits. A large body of empirical literature has established a clear link between poor housing conditions and poor health [77]. There are multiple relationships between the diverse characteristics of housing environments that can positively or negatively influence physical and mental health [11]. These controversies show that due to the complexity of living conditions and the diversity of living spaces, the mechanism of the impact of living conditions on health has not been clarified so far, and a general consensus has not yet been reached. The relationship between the two is not clear at a glance. The results of this study indicate that people living in high-quality housing are more likely to have lower self-rated health. For this conclusion, two points need to be emphasized. First, this study actually compared two types of residents living in bungalows, Siheyuans, units, villas, townhouses, and small buildings, and found that the latter’s self-rated health is lower than the former. The latter are mainly wealthy people. According to the “2017 Hurun Wealth Report” published by the Hurun Research Institute, over 60% of richest people in China have “sub-healthy” symptoms. In other words, the health of the wealthy people living in high-quality houses such as villas, townhouses, and small buildings will be worse than those of ordinary people living in bungalows, Siheyuans, and units. Second, this research is about self-reported health, not objective health. Self-reported health will be affected to a certain extent by social and psychological factors, and wealthy people living in high-quality housing are more likely to report low self-rated health. For example, according to the “2017 White Paper on the Health Index of China’s High Net Worth People”, 80% of the wealthy in China believe that their health is not good.

There are many reasons for this. For example, wealthy people living in high-quality housing have higher expectations of health, are more likely to fail to meet their existing health conditions, and will rate their health lower [58,61]. Rich people living in high-quality housing are more stressed, which is not good for their health [62]. Therefore, the impact of housing conditions on health should be analyzed dialectically. Of course, there are differences in national conditions and cultures between countries. Therefore, the results obtained in this study based on data from China may be different from the results obtained using data from other countries. Therefore, in the interpretation of the results, it is necessary to pay attention to the cultural differences between different countries and regions.

In addition, keeping the house clean and tidy is also important to health. The results of this study show that the self-rated health of people with tidy homes is higher than that of people with untidy homes. The results of this study are consistent with those of previous studies [67,68]. Clean and tidy homes involve extensive housework and physical activity. One study reported that daily activities such as housework can not only increase happiness, but also activate the brain and increase one’s energy [78]. The self-discipline and organizational abilities of people who are tidy at home are also strong, and these qualities have a promoting effect on health [79]. Therefore, for those who cannot live in spacious and bright high-quality houses, as long as they can develop good housekeeping habits and keep their living space clean and tidy, they can also live a healthy life.

In addition, the results of this study indicate that the type of community does not have a significant impact on self-rated health. This research also shows that the older a person is, the worse their self-rated health. This is consistent with the results of previous studies [80,81]. Moreover, this research shows that the self-rated health of men is better than that of women; that of those with a spouse is worse than that of those without a spouse; that of those with a high degree of education is better than those with a low degree; and that of those with a higher income is higher than those with a lower income. It can be seen that both individual demographics and socio-economic factors have an impact on SRH.

This study contributes to the extant literature in three ways. First, it extends understanding of the association between living conditions and health. Second, it extends the social determinants of health theory by drawing insights obtained from a daily living lens. Third, this study found that improvement in housing conditions does not necessarily lead to an improvement in self-rated health. This finding does not coincide with the overwhelming majority of the literature on this topic. The existence of this kind of counterexample shows that the relationship between housing conditions and health is complex and non-linear. Therefore, it helps to eliminate people’s mindset and help people to objectively and comprehensively evaluate the impact of housing conditions on health. These findings have important practical guiding value for the formulation of relevant policies.

This study found that there is an important correlation between living conditions and health status, which is consistent with most previous research conclusions. However, due to the content of the questionnaire and the limitations of the survey, this research has the following limitations. First, due to the limitations of the data, the living condition indicators investigated in this research are limited, and other living condition factors may be omitted. Second, because of the attributes of cross-sectional data, it is difficult to reveal the causal mechanism of living conditions on health. Third, for the measurement of health, this study uses subjective self-rated health, and lacks objective indicators of health. In addition, the meaning of living conditions is becoming increasingly complex, and there are often interactions with personal characteristics. Thus, the relationship between living conditions and health needs further study.

## 6. Conclusions

This study used CFPS2018 data to empirically study the impact of living conditions on self-rated health. The study found that the following living conditions affected the self-rated health of residents: the use of sanitary water sources, the use of clean fuels, complete housing ownership, and cleanliness of ordinary houses are all associated with better self-rated health. In addition, gender, age, marital status, education level, and income all have a significant impact on the health of the population.

Based on the above conclusions, in order to improve residents’ health, this article has the following policy suggestions: First, it is necessary to strengthen water source management and increase the penetration rate of tap water to ensure the safety of water for the majority of people. Second, it is necessary to promote and increase the household penetration rate of clean fuels. Third, the government should increase housing security investment to allow more families to own their houses, enhancing their sense of belonging, security, and happiness. Fourth, it is necessary to increase housing hygiene and safety publicity to strengthen people’s sense of responsibility, maintain home cleanliness and hygiene, and build a clean and beautiful home environment.

## Figures and Tables

**Table 1:** Variable definitions.

Variable	Specific Explanation
Explained Variable	
Self-rated health	0 means unhealthy, 1 means healthy (0 = normal, unhealthy, 1 = relatively healthy, very healthy, very healthy)
Explanatory variables	
Cooking water	0 means unsanitary raw water (rivers and lakes, well water, rainwater, cellar water, pond water/spring water, others); 1 means sanitary water (tap water, bottled water/pure water/filtered water)
Cooking fuel	0 refers to traditional fuel (firewood, coal, canned gas/liquefied gas, others); 1 refers to clean fuel (natural gas/pipeline gas, solar energy/biogas, electricity)
Housing ownership	0 = family members have partial ownership; 1 = family members have full ownership
Housing Type	0 refers to ordinary residences (bungalows, Siheyuans, units, others); 1 refers to high-quality residences (villas, townhouses, small buildings)
Home tidiness	The original data have 7 categories, where the larger the value, the higher the degree. Converted into a binary variable: 0 = 1, 2, 3, 4, indicating that the home is dirty; 1 = 5, 6, 7, indicating that the home is tidy
Control variables	
Community type	0 = Village committee, indicating that the infrastructure is relatively backward; 1 = neighborhood committee, indicating that the infrastructure is relatively up-to-date
Age	Continuous variable, the value range is between 16 and 95 years old
Gender	0 = female, 1 = male
Marital status	0 = unmarried, divorced, widowed, meaning no spouse, 1 = spouse (married), cohabiting, meaning there is a spouse
Education	There are 7 categories of raw data: illiterate/semi-literate, elementary school, junior high school, high school/technical school/vocational high school, college, university, undergraduate, master’s, and doctoral. Converted into a binary variable: 0 = illiterate/semi-literate, meaning not educated, 1 = other six categories, meaning educated
Income at the local level	The original data has 5 categories. The larger the value, the higher the income relative to the local. Converted into a binary variable: 0 = 1, 2, 3, which means lower wages; 1 = 4, 5, which means higher wages

**Table 2:** Chi-square contingency table (*N* = 22,710).

Variable	Sample Size *n* (%)	Health Status		χ^2^ (*p*)
		Healthy *n* (%)	Not Healthy *n* (%)	
Cooking water				
sanitary water	16,602 (73.1)	11,365 (74.3)	5237 (70.6)	34.64 *** (0.000)
unsanitary water	6108 (26.9)	3929 (25.7)	2179 (29.4)	
Cooking fuel				
clean fuel	8853 (39.0)	6116 (40.0)	2737 (36.9)	19.96 *** (0.000)
traditional fuel	13,857 (61.0)	9178 (60.0)	4679 (63.1)	
Housing ownership				
complete ownership	20,053 (88.3)	13,576 (88.8)	6477 (87.3)	9.87 *** (0.002)
incomplete ownership	2657 (11.7)	1718 (11.2)	939 (12.7)	
Housing type				
high-quality housing	5975 (26.3)	3995 (26.1)	1980 (26.7)	0.86 (0.354)
ordinary housing	16,735 (73.7)	11,299 (73.9)	5436 (73.3)	
Home tidiness				
tidy home	15,079 (66.4)	10,494 (68.6)	4585 (61.8)	103.18 *** (0.000)
messy home	7631 (33.6)	4800 (31.4)	2831 (38.2)	
Community type				
neighborhood committee	5938 (26.1)	4111 (26.9)	1827 (24.6)	13.02 *** (0.000)
village committee	16,772 (73.9)	11,183 (73.1)	5589 (75.4)	
Gender				
Male	11,296 (49.7)	8071 (52.8)	3225 (42.5)	172.24 *** (0.000)
Female	11,414 (50.3)	7223 (47.2)	4191 (56.5)	
Marital status				
with spouse	19,047 (83.9)	12,684 (82.9)	6363 (85.8)	30.34 *** (0.000)
without spouse	3663 (16.1)	2610 (17.1)	1053 (14.2)	
Education				
Educated	16,996 (74.8)	12,280 (80.3)	4716 (63.6)	739.76 *** (0.000)
Uneducated	5714 (25.2)	3014 (19.7)	2700 (36.4)	
Income at the local level				
higher wages	5446 (24.0)	3852 (25.2)	1594 (21.5)	37.45 *** (0.000)
lower wages	17,264 (76.0)	11,442 (74.8)	5822 (78.5)	
Health status				
Healthy	15,294 (67.3)	-	-	-
not healthy	7416 (32.7)	-	-	

Note: *** *p* ≤ 0.01.

**Table 3:** Multivariable logistic regression analysis (*N* = 22,710).

Variable	Odd Ratio	SE	95% CI
Cooking water: sanitary vs. unsanitary water	1.159 ***	0.035	1.082–1.241
Cooking fuel: clean fuel vs. traditional fuel	1.141 **	0.033	1.066–1.231
Housing ownership: complete ownership vs. incomplete ownership	1.115 **	0.046	1.018–1.221
Type of housing: high-quality housing vs. ordinary housing	0.908 ***	0.034	0.849–0.971
Home tidiness: tidy home vs. messy home	1.261 ***	0.031	1.186–1.342
Community type: neighborhood vs. village	1.035	0.039	0.959–1.117
Age: continuous variable	0.964 ***	0.001	0.962–0.966
Gender: male vs. female	1.452 ***	0.031	1.368–1.542
Marital status: with spouse vs. without spouse	0.868 ***	0.043	0.797–0.945
Education: educated vs. uneducated	1.438 ***	0.036	1.339–1.544
Income at local level: higher wages vs. lower wages	1.558 ***	0.036	1.451–1.672
constant	6.212 ***	0.093	
Chi-square (sig.)	2240.664 (0.000)		
−2Log likelihood	26451.604		
Cox and Snell *R*^2^	0.194		
ρ^2^ (Nagelkerke)	0.191		

Note: ** *p* ≤ 0.05; *** *p* ≤ 0.01.

## Data Availability

The raw data of CFPS were used for this study with permission from the Institute of Social Survey Study, Peking University (ISSS).

## Appendix A

**Table A1:** Descriptive statistics and correlation of variables.

Variable	M	S.D.	1	2	3	4	5	6	7	8	9	10	11	12
1. Health	0.673	0.469	--											
2. Water	0.731	0.443	0.039 ***	--										
3. Fuel	0.390	0.488	0.03 ***	0.194 ***	--									
4. Housing Ownership	0.883	0.321	0.021 ***	−0.061 ***	−0.046 ***	--								
5. Housing Type	0.263	0.440	−0.006	−0.023 ***	−0.068 ***	0.053 ***	--							
6. Tidy	0.664	0.472	0.067 ***	0.041 ***	0.08 ***	0.008	0.055 ***	--						
7. Community	0.262	0.439	0.024 ***	0.306 ***	0.338 ***	−0.141 ***	−0.135 ***	0.103 ***	--					
8. Age	49.661	15.792	−0.267 ***	−0.007	−0.011	−0.03 ***	−0.046 ***	−0.054 ***	0.025 ***	--				
9. Gender	0.497	0.500	0.087 ***	−0.011	−0.013 **	0.007	0.001	−0.018 **	−0.014 **	0.011	--			
10. Marriage	0.839	0.368	−0.037 ***	−0.007	−0.008	0.032 ***	0.014 **	0.031 ***	−0.018 ***	0.142 ***	−0.009	--		
11. Education	0.748	0.434	0.18 ***	0.078 ***	0.132 ***	−0.011	0.016 **	0.089 ***	0.168 ***	−0.363 ***	0.186 ***	0.015 **	--	
12. Income	0.240	0.427	0.041 ***	−0.01	−0.036 ***	0.025 ***	−0.02 ***	−0.007	−0.061 ***	0.108 ***	−0.025 ***	0.02 ***	−0.121 ***	--

Note: *** *p* ≤ 0.01, ** *p* ≤ 0.05.
